# Supplementary material for: Observation of exceptional point in a PT broken non-Hermitian system simulated using a quantum circuit
Source: Sci Rep. 2021 Jul 5;11:13795. doi: 10.1038/s41598-021-93192-x (PMC8257716; doi:10.1038/s41598-021-93192-x)
Supplement: Supplementary file 1 — Supplementary Information. [file 41598_2021_93192_MOESM1_ESM.pdf]

SUPPLEMENTARY INFORMATION
for
“Observation of exceptional point in a PT broken non-Hermitian system simulated using a quantum circuit”

Geng-Li Zhang,[1, 2, 3] Di Liu,[4, 5] and Man-Hong Yung[1, 6, 7, 8, *]

[1] *Central Research Institute, Huawei Technologies, Shenzhen 518129, China*

[2] *Department of Physics, The Chinese University of Hong Kong, Shatin, N. T., Hong Kong, China*

[3] *Center for Quantum Coherence, The Chinese University of Hong Kong, Shatin, N.T., Hong Kong, China*

[4] *Key Laboratory of Quantum Information, CAS, University of Science and Technology of China, Hefei, Anhui 230026, China*

[5] *Synergetic Innovation Center of Quantum Information & Quantum Physics, University of Science and Technology of China, Hefei, Anhui 230026, China*

[6] *Department of Physics, Southern University of Science and Technology*

[7] *Shenzhen Institute for Quantum Science and Engineering, Southern University of Science and Technology, Shenzhen 518055, China*

[8] *Guangdong Provincial Key Laboratory of Quantum Science and Engineering, Shenzhen Institute for Quantum Science and Engineering, Southern University of Science and TechnologyShenzhen 518055, GuangdongChina*

[*] yung@sustech.edu.cn

I. USEFUL FUNCTIONS OF THE SIMULATOR BACKEND

Several functions in projectQ/HiQ are utilized in the simulator backend, which simplified the simulation of the circuit. But, to be noticed, they are only available to the simulator backend and cannot be used for simulations on real quantum chips.

The `cheat` function can be used to directly access and manipulate the full wavefunction. This function returns a list of two elements. `cheat()[0]` is the mapping of the qubits with the bitlocations, which may depend on how the compiler is optimized. `cheat()[1]` is the amplitudes of the wavefunction. they are stored as a `numpy` array of length 2^n with n being the number of qubits.

The `set_wavefunction` function is used to set the qubits to a specific state. This can be used a debugging tool, for instance, to verify the correctness the non-Hermitian units.

The `collapase_wavefunction` can be used to directly obtain the desired post-selected wavefunction by specifying a specific measurement outcome (unless the probability is 0), e.g., $|0\rangle$.

The following code fragment illustrates the usage of these functions:

```
eng.flush()
# set the initial wavefunction
eng.backend.set_wavefunction(psi_qbv[0], qubit)
H | qubit
ancilla = eng.allocate_qubit()
C(Rx(phi)) | (qubit, ancilla)


# the wavefunction when the ancilla is at 0
eng.flush()
eng.backend.collapse_wavefunction(ancilla, [0])
# deallocate the ancilla
del ancilla
eng.flush()
# print the wavefunction
print(eng.backend.cheat()[1])
```

where the `flush()` function is required to push all of above gates to the simulator and execute.

II. OSCILLATING FOR $\Gamma < \theta$

When $\Gamma < \theta$, the eigenvalues and eigenvectors can be recast as

$$\lambda_{\pm} = -\frac{i\Gamma}{2} \pm \frac{\theta}{2}\cos\alpha, \quad |v_{\pm}\rangle = \frac{1}{\sqrt{2}}\begin{bmatrix} \pm e^{\pm i\alpha} \\ 1 \end{bmatrix}, \tag{S1}$$

where $\alpha = \sin^{-1}(\Gamma/\theta)$. The computational states can be written in eigenbasis:

$$\begin{cases} |0\rangle = \dfrac{1}{\sqrt{2}\cos\alpha}\left(|v_+\rangle - |v_-\rangle\right), \\ |1\rangle = \dfrac{\sqrt{2}}{1+e^{i2\alpha}}\left(|v_+\rangle + e^{i2\alpha}|v_-\rangle\right). \end{cases} \tag{S2}$$

Starting from the initial $|0\rangle$ state, the state after the evolution is

$$\begin{aligned} |\psi^{(0)}(t)\rangle &= \frac{1}{\sqrt{2}\cos\alpha}\left(e^{-i\lambda_+ t}|v_+\rangle - e^{-i\lambda_- t}|v_-\rangle\right) \\ &= \frac{e^{-\Gamma t/2}}{\sqrt{2}\cos\alpha}\left(e^{-i\frac{\theta t}{2}\cos\alpha}|v_+\rangle - e^{i\frac{\theta t}{2}\cos\alpha}|v_-\rangle\right), \end{aligned} \tag{S3}$$

from which we can get the expectation value

$$M_z^{(0)}(t) = \frac{\cos^2(\frac{\theta t}{2}\cos\alpha - \alpha) - \sin^2(\frac{\theta t}{2}\cos\alpha)}{\cos^2(\frac{\theta t}{2}\cos\alpha - \alpha) + \sin^2(\frac{\theta t}{2}\cos\alpha)}. \tag{S4}$$

Similarly, starting from the initial $|1\rangle$ state, the expectation value is

$$M_z^{(1)}(t) = \frac{\sin^2(\frac{\theta t}{2}\cos\alpha) - \cos^2(\frac{\theta t}{2}\cos\alpha + \alpha)}{\sin^2(\frac{\theta t}{2}\cos\alpha) + \cos^2(\frac{\theta t}{2}\cos\alpha + \alpha)}. \tag{S5}$$

Therefore starting from the initial completely mixed state $\rho(0) = I/2$, the expectation value $M_z(t) = (M_z^{(0)}(t) + M_z^{(1)}(t))/2$ is always oscillating and hence has no stationary point. For instance, when $\theta \gg \Gamma$, $\alpha \approx \Gamma/\theta$, $\cos\alpha \approx 1 - \Gamma^2/2\theta^2$, and at the long time limit ($t \gg 1/\theta$),

$$\begin{aligned} M_z(t) &\approx \frac{1}{4}\left[\cos(\theta t\cos\alpha - 2\alpha) - \cos(\theta t\cos\alpha + 2\alpha)\right] \\ &= \frac{1}{2}\sin(\theta t\cos\alpha)\sin(2\alpha) \\ &\approx \frac{1}{2}\sin(2\alpha)\sin(\theta t), \end{aligned} \tag{S6}$$

which means that the long time average of the expectation value is 0.

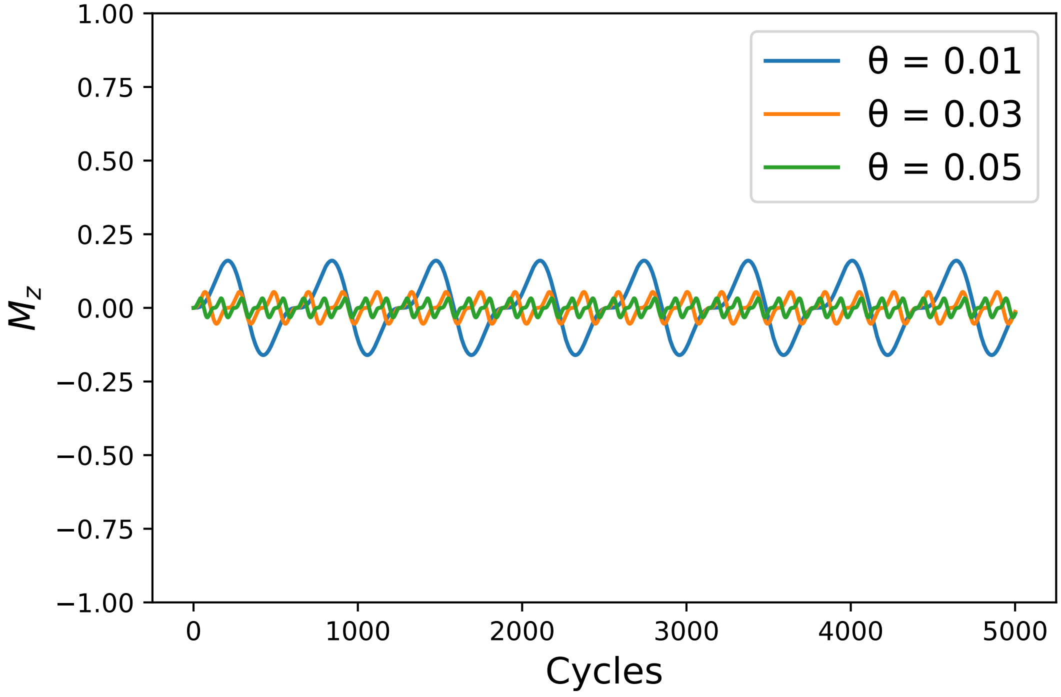

FIG. S1. M_z Oscillates for $\Gamma < \theta$, and ϕ is fixed at 0.1. The results are simulated with `collapse_wavefunction` and `cheat` functions of the simulator backend.The expectation values are always oscillating, and the long time average is vanishing.
